# Machine-Learning-Guided Design of Nanostructured Metal Oxide Photoanodes for Photoelectrochemical Water Splitting: From Material Discovery to Performance Optimization

**DOI:** 10.3390/nano15120948

**Published:** 2025-06-18

**Authors:** Xiongwei Liang, Shaopeng Yu, Bo Meng, Yongfu Ju, Shuai Wang, Yingning Wang

**Affiliations:** 1 Cold Region Wetland Ecology and Environment Research Key Laboratory of Heilongjiang Province, Harbin University, Harbin 150086, China; liangxiongwei007@163.com (X.L.); mengbomune@aliyun.com (B.M.); juyongfu@163.com (Y.J.); okdashuai@126.com (S.W.); 14b927013@hit.edu.cn (Y.W.); 2 State Key Laboratory of Urban Water Resource and Environment, Harbin Institute of Technology, Harbin 150086, China

**Keywords:** photoelectrochemical water splitting, nanostructured photoanodes, metal oxide semiconductors, machine learning, materials informatics, solar fuels

## Abstract

The rational design of photoanode materials is pivotal for advancing photoelectrochemical (PEC) water splitting toward sustainable hydrogen production. This review highlights recent progress in the machine learning (ML)-assisted development of nanostructured metal oxide photoanodes, focusing on bridging materials discovery and device-level performance optimization. We first delineate the fundamental physicochemical criteria for efficient photoanodes, including suitable band alignment, visible-light absorption, charge carrier mobility, and electrochemical stability. Conventional strategies such as nanostructuring, elemental doping, and surface/interface engineering are critically evaluated. We then discuss the integration of ML techniques—ranging from high-throughput density functional theory (DFT)-based screening to experimental data-driven modeling—for accelerating the identification of promising oxides (e.g., BiVO_4_, Fe_2_O_3_, WO_3_) and optimizing key parameters such as dopant selection, morphology, and catalyst interfaces. Particular attention is given to surrogate modeling, Bayesian optimization, convolutional neural networks, and explainable AI approaches that enable closed-loop synthesis-experiment-ML frameworks. ML-assisted performance prediction and tandem device design are also addressed. Finally, current challenges in data standardization, model generalizability, and experimental validation are outlined, and future perspectives are proposed for integrating ML with automated platforms and physics-informed modeling to facilitate scalable PEC material development for clean energy applications.

## 1. Introduction

The accelerating global demand for carbon-neutral energy systems has intensified the development of solar-driven technologies for clean hydrogen production [1,2,3,4]. Among these, photoelectrochemical (PEC) water splitting is a particularly attractive route, directly converting sunlight and water into molecular hydrogen and oxygen using semiconductor photoelectrodes [5,6,7,8]. As a scalable and sustainable alternative to fossil-fuel-derived hydrogen, PEC systems promise decentralized solar-to-fuel conversion. However, their efficiency and long-term operational stability remain limited by materials constraints, particularly the performance of the photoanode, which governs the sluggish oxygen evolution reaction (OER) [9,10,11,12].

Metal oxide semiconductors, such as TiO_2_, α-Fe_2_O_3_ (hematite), WO_3_, and BiVO_4_, are the most extensively investigated photoanode materials owing to their natural abundance, resistance to photocorrosion, and structural versatility [13,14,15]. Yet, each presents its own limitations. TiO_2_ is highly stable but has a large bandgap (~3.2 eV), restricting visible light absorption [16]. Hematite absorbs visible light (~2.1 eV) but exhibits poor charge transport and a short hole diffusion length (<5 nm). BiVO_4_ offers a near-ideal bandgap (~2.4 eV) and favorable band edge positions, but its low electron mobility and rapid surface recombination limit charge extraction [17]. Consequently, intensive research has been devoted to tuning their properties through doping, nanostructuring, heterojunction formation, and surface catalytic modifications [18,19,20,21,22].

Despite these advances, traditional materials discovery in PEC systems remains slow and empirical. The multidimensional optimization space—including composition, crystallinity, defect states, surface energetics, morphology, and interfacial chemistry—renders trial-and-error methods inefficient and often biased by intuition. Furthermore, the coupling between bulk and interfacial processes in photoanodes introduces nonlinear dependencies that are difficult to decouple experimentally [23]. These challenges call for accelerated, data-driven design approaches that can systematically explore complex parameter spaces and reveal hidden correlations among structure, properties, and performance [24].

Machine learning (ML), with its capability to model nonlinear relationships and extract patterns from high-dimensional datasets, has emerged as a powerful tool in materials science [25]. In PEC research, ML has enabled rapid prediction of key properties—such as bandgap, formation energy, stability, carrier mobility, and oxygen vacancy formation energy—based on composition or crystal structure descriptors [26]. ML models trained on data from open materials databases like the Materials Project, OQMD, and AFLOW [27] have demonstrated impressive accuracy and transferability in screening large compositional spaces. For example, crystal graph convolutional neural networks (CGCNNs) can directly learn from structural graphs and predict formation energy and bandgap without manual feature engineering [28].

Recent applications of ML in PEC include prediction of dopant effects in BiVO_4_ and Fe_2_O_3_ photoanodes, estimation of PEC performance from SEM morphology using convolutional neural networks, and optimization of catalyst loading using Bayesian frameworks [29,30]. These studies illustrate the shift from passive prediction to active learning and inverse design, where ML guides synthesis planning, experimental prioritization, and feedback control in closed-loop experimental systems [29]. Moreover, explainable ML techniques such as SHAP (Shapley Additive Explanations) are increasingly used to interpret model outputs, helping researchers identify dominant features like electronegativity difference or ionic radius mismatch that influence PEC activity [31,32,33].

Nonetheless, challenges persist. Most ML models rely on limited or inconsistent data, often derived under varying experimental protocols. Furthermore, generalizability across materials classes, as well as model interpretability and integration with physical laws, remain active areas of development [34,35,36]. To fully realize ML’s potential in PEC materials design, future efforts must focus on creating standardized datasets, incorporating physics-informed learning architectures, and coupling ML predictions with high-throughput synthesis and characterization platforms [35].

In this review, we examine how machine learning is reshaping the discovery and optimization of nanostructured metal oxide photoanodes for PEC water splitting. We begin by revisiting the physicochemical requirements for ideal photoanode materials, then survey conventional enhancement strategies. We then provide a comprehensive overview of ML models and workflows applied to PEC materials—from data curation and feature representation to predictive modeling and experimental integration. Finally, we highlight opportunities for closed-loop design systems, explainable AI, and the fusion of ML with first-principles simulations to advance the next generation of solar fuel technologies.

## 2. Fundamentals of Nanostructured Metal Oxide Photoanodes

### 2.1. Operational Principles and Material Requirements

**Figure 1:**
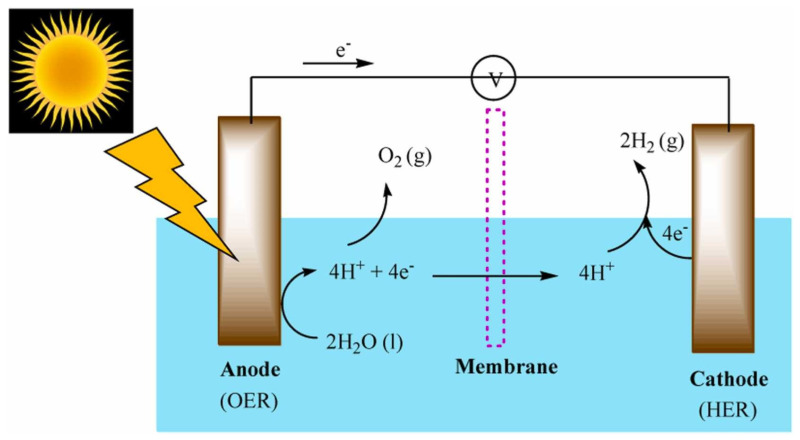
Schematic illustration of a photoelectrochemical (PEC) water splitting cell [41]. Copyright, 2024 Elsevier.

In a PEC water splitting cell, the photoanode semiconductor must absorb sunlight and generate electron–hole pairs with sufficient energy to drive the OER at its surface [37,38]. This requires a bandgap ≥ 1.23 eV (the thermodynamic minimum for water splitting) and appropriate band edge alignment such that the valence band maximum is below the O_2_/H_2_O oxidation potential (≈+1.23 V vs. RHE) and the conduction band minimum is above the H^+^/H_2_ reduction potential (≈0 V vs. RHE) [38]. Efficient solar utilization further demands a bandgap in the visible range (~1.8–2.5 eV) to harness a large portion of the solar spectrum [39]. Additionally, the photoanode material should exhibit long minority carrier (hole) diffusion lengths or be structured such that photogenerated holes can reach the surface OER sites before recombining in the bulk [39]. Fast majority carrier (electron) transport to the back contact is equally important to minimize resistive losses. Stability under illumination and in electrolyte (often harsh pH) is a critical requirement—many narrow-bandgap semiconductors photocorrode during water oxidation, which is why robust oxides are heavily studied [39,40]. Given these stringent criteria, only a few metal oxides have emerged as feasible photoanodes, each with distinct advantages and limitations, as summarized below. The overall mechanism is illustrated in Figure 1, where photogenerated electrons reduce H^+^ to H_2_ at the cathode, and holes drive the OER at the photoanode. Ion exchange occurs across a membrane, which also prevents mixing of evolved gases [41].

### 2.2. TiO_2_ (Titanium Dioxide)

TiO_2_ was the first demonstrated PEC photoanode and remains a benchmark for stability. It has a wide bandgap (3.0–3.2 eV, rutile/anatase), absorbing only UV (~5% of solar spectrum). While TiO_2_’s conduction and valence band positions straddle the water redox levels (enabling unassisted water splitting under UV), its wide bandgap severely limits achievable photocurrent under sunlight [39,42]. TiO_2_ is often employed in tandem cells as a protective layer or UV-active component rather than as a standalone visible-light photoanode. Strategies to improve TiO_2_’s performance include doping with anions or cations (e.g., N, C, Fe) to slightly narrow the bandgap or introduce mid-gap states for visible light absorption [29,42], and nanostructuring to improve charge separation. For example, highly ordered TiO_2_ nanotube arrays offer large surface area and short hole diffusion distances, enhancing photocurrents under UV illumination [42]. However, excessive doping of TiO_2_ can introduce recombination centers, and achieving significant visible-light activity remains challenging. Overall, TiO_2_’s extreme stability (it resists photocorrosion even in strong acidic or basic electrolytes) [40] makes it valuable as a model system and protective layer, but its large bandgap keeps its standalone solar-to-hydrogen (STH) conversion efficiency low (theoretical max ~1–2%) [39].

TiO_2_, with a wide bandgap (~3.2 eV), primarily absorbs ultraviolet photons. Metal doping (Fe, Cr, Ni) introduces shallow mid-gap states, enhancing visible light response and temporarily trapping carriers to reduce recombination losses. Integrating TiO_2_ into heterojunctions (e.g., with BiVO_4_ or WO_3_) provides internal electric fields, significantly promoting directional electron–hole separation and enhancing overall charge collection efficiency.

### 2.3. α-Fe_2_O_3_ (Hematite)

Hematite is an appealing photoanode material with a bandgap ~2.1 eV, theoretically capable of ~12.6 mA cm^−2^ photocurrent under AM 1.5 G (corresponding to STH ~15%) [39,43]. It is inexpensive, stable in alkaline electrolyte, and absorbs a broad portion of visible light (up to ~590 nm). The main drawbacks of Fe_2_O_3_ are its short hole diffusion length (2–4 nm) and poor electrical conductivity due to polaronic charge transport. Consequently, bulk hematite suffers severe charge recombination; planar films yield low photocurrent (<1 mA cm^−2^) and require a large anodic bias (~0.8 V vs. RHE) to drive appreciable water oxidation [39]. Nanostructuring hematite is essential: by using nanowires, ultrathin films, or porous nanostructures, photogenerated holes are created within a few nanometers of the electrolyte interface, improving their odds of reaching the surface [39,44]. For instance, nano-porous hematite films doped with silicon were shown to significantly enhance charge separation and photocurrent. Si doping (at a few at. %) increases electron conductivity by introducing donor states and reduces surface recombination by altering surface states. In one study, nanostructured Si-doped hematite achieved ~2 mA cm^−2^ at 1.23 V_RHE, about an order of magnitude higher than undoped flat hematite. Sn doping is another effective strategy: Sn^4+^ substitutes for Fe^3+^, increasing electron density and conductivity—Sn-doped hematite nanorods yielded higher photocurrents and a negatively shifted onset potential [45]. Despite these improvements, hematite’s performance is still limited by sluggish water oxidation kinetics at the surface and a high minority carrier injection barrier. The addition of OER co-catalysts such as FeOOH/NiOOH can dramatically lower the onset potential and boost the photocurrent of hematite [46]. For example, ultrathin hematite decorated with a NiOOH layer showed a notable cathodic shift in onset (by ~0.2–0.3 V) due to improved surface hole transfer kinetics [46]. State-of-the-art hematite photoanodes (employing nanostructuring, ~5% dopant incorporation, and catalyst coating) can achieve ~4–5 mA cm^−2^ at 1.23 V_RHE in strong base, but still fall short of the theoretical limit [39]. The charge transport and recombination issues inherent to hematite impose a performance ceiling that has proven difficult to overcome [44].

α-Fe_2_O_3_ possesses an optimal narrow bandgap (~2.1 eV) for visible-light absorption, yet its performance is limited by low electron mobility due to polaronic conduction and short hole diffusion lengths. Introducing donor doping (Sn^4+^ or Ti^4+^) improves electrical conductivity, reduces bulk resistance, and facilitates charge migration. Surface modification with NiOOH or FeOOH catalysts further accelerates hole transfer kinetics, effectively suppressing recombination pathways.

### 2.4. BiVO_4_ (Bismuth Vanadate)

BiVO_4_ has emerged as one of the most promising photoanode materials in the past decade [47]. It possesses a near-ideal bandgap (~2.4 eV, monoclinic scheelite phase) that yields a theoretical photocurrent of 7.5 mA cm^−2^ (STH ~9.2%) under 1 sun [47]. BiVO_4_’s band edges are well-positioned: the conduction band (0 V vs. RHE) is just sufficient for H^+^ reduction (hence it needs an external bias or a dual-absorber system for unassisted water splitting), and the valence band (+2.4 V vs. RHE) provides a strong driving force for OER. BiVO_4_ is an n-type semiconductor with moderate hole diffusion length (~70–100 nm) and poor electron transport due to low mobility (electron transport is a rate-limiting step) [47,48]. To maximize performance, researchers have employed a combination of nanostructuring, doping, and catalytic surface layers. Early studies found that Mo or W doping (a few percent) in BiVO_4_ markedly improves electron conductivity by introducing donor states that fill electron traps, thereby enhancing photocurrent [47,48]. For instance, W-doped BiVO_4_ showed a two- to three-fold increase in photocurrent compared to undoped BiVO_4_ under illumination [48]. Nanostructured architectures like nanoporous or dendritic BiVO_4_ thin films further improved charge separation by creating a large internal surface within minority carrier diffusion distance [46]. A landmark achievement was reported by Kim and Choi, who combined a nanoporous Mo-doped BiVO_4_ photoanode with sequential FeOOH/NiOOH co-catalyst layers [46]. This dual-layer catalyst strategy dramatically reduced surface recombination; the BiVO_4_/FeOOH/NiOOH photoanode attained a photocurrent of 2.73 mA cm^−2^ at a mere 0.6 V_RHE (and ~4.2 mA cm^−2^ at 1.23 V_RHE) [46], which was a record at the time. More recently, innovative co-doping and interface engineering have pushed BiVO_4_ performance even higher. Yang et al. demonstrated an Fe–N co-doped BiVO_4_ in conjunction with a tailored FeNiOOH surface catalyst, achieving an astonishing 7.0 mA cm^−2^ at 1.23 V_RHE under one-sun illumination [29]. Separately, a BiVO_4_ photoanode coupled with a phosphorus-oxygen bonded FeNi hydroxide catalyst exhibited 6.7 mA cm^−2^ at 1.23 V_RHE with greatly enhanced stability [49]. These results approach the theoretical limit for BiVO_4_ and underscore the importance of interface chemistry: the strong electronic coupling and favorable band alignment at the BiVO_4_/catalyst interface facilitated efficient hole transfer and suppressed photocorrosion [29,49]. BiVO_4_ does require an external bias (~0.6 V in the best systems) to drive water splitting, so it is often used in bias-free tandem designs with a photocathode or photovoltaic. Nevertheless, its ease of synthesis and continually improving performance make BiVO_4_ a model system to test new ideas in photoanode engineering.

BiVO_4_ features an ideal bandgap (~2.4 eV) and significant hole diffusion length but suffers from intrinsically poor electron mobility. Incorporating W^6+^ or Mo^6+^ dopants significantly mitigate intrinsic defects, enhancing carrier density and electrical conductivity. Additionally, heterostructure with tungsten oxide (WO_3_) efficiently funnels electrons, while dual-layer FeOOH/NiOOH surface catalysts rapidly extract holes, driving efficient multi-electron oxygen evolution reactions.

### 2.5. WO_3_ (Tungsten Trioxide)

Tungsten trioxide (WO_3_) is another extensively studied photoanode oxide. It has a moderate bandgap (~2.6–2.8 eV) and excellent photochemical stability (especially in acidic electrolytes), which allows it to absorb a portion of visible light and resist photocorrosion [50,51]. However, WO_3_’s conduction band minimum lies below the H^+^/H_2_ potential, meaning that WO_3_ alone requires a significant applied bias (or coupling with a higher-energy absorber) to drive water splitting [52,53]. In practice, WO_3_ is often employed in heterojunctions: for example, a thin WO_3_ underlayer beneath a BiVO_4_ absorber forms an efficient type-II junction [54]. In this configuration, WO_3_ collects electrons generated in BiVO_4_ and conducts them to the back contact, while holes remain in BiVO_4_ for oxygen evolution. Such a BiVO_4_/WO_3_ heterojunction was shown to significantly improve the overall photocurrent and photovoltage compared to either material alone [55,56]. Similar strategies use WO_3_ in combination with narrow-gap absorbers (or protective coatings) to exploit its stability while compensating for its low reduction potential. Despite its limitations, WO_3_’s robustness and synergies in multi-layer electrodes justify its inclusion among the key oxide photoanodes [57].

WO_3_ characterized by a moderate bandgap (~2.7 eV), exhibits favorable electron transport properties and exceptional photoelectrochemical stability. It functions effectively as an electron transfer backbone within composite heterojunction systems. Coupling WO_3_ with semiconductors like BiVO_4_ or TiO_2_ enhances charge separation efficiency via energy-band engineering, markedly suppressing charge recombination and improving PEC performance.

### 2.6. Other Noteworthy Oxide Photoanodes

A few other oxides and related compounds merit brief mention. ZnO has a bandgap (~3.2 eV) similar to TiO_2_ and can serve as a photoanode under UV illumination, but it suffers from anodic dissolution in neutral or acidic electrolytes, limiting its practical use [58]. With surface modification or alkaline conditions, ZnO nanorods have shown respectable UV PEC performance, though instability remains an issue [58]. Tungsten bronze oxides (e.g., Sr_2_Fe_2−x_W_x_O_6_) and complex metal oxides—such as delafossites and perovskite-type oxides—have been explored via combinatorial studies. While some perovskite oxides suffer from wide bandgaps or instability, recent research has demonstrated that optimized perovskite oxide electrodes can achieve high-performance photoelectrochemical (PEC) water splitting, due to their tunable electronic structure and catalytic versatility [38,59,60]. More recently, non-oxide photoanodes such as Ta_3_N_5_ and other oxynitrides have gained attention for their narrower bandgaps (~2.1 eV) and strong visible absorption [59]. Ta_3_N_5_ photoanodes can achieve high photocurrents (~5–7 mA cm^−2^) when properly protected and catalyzed but are prone to self-oxidation without careful surface treatments. While this review focuses on oxides, it is worth noting that insights from oxides often inform the development of oxynitrides or sulfide photoelectrodes, and vice versa.

## 3. Nanostructuring, Doping, and Interface Engineering—Conventional Approaches

### 3.1. Nanostructuring

Engineering nanoscopic architectures—nanowires, nanotubes, mesoporous scaffolds, or ultrathin flakes—places every photogenerated hole within its diffusion length of the semiconductor/electrolyte interface, thereby maximising minority-carrier collection. Concomitantly, the enlarged geometric surface area affords a higher density of catalytically active sites. For example, transforming a dense BiVO_4_ film into a three-dimensionally interconnected nanoporous network nearly doubles the steady-state O_2_-evolution current, an improvement that can be ascribed to shortened hole-transport pathways and enhanced electrolyte contact [46]. Analogous gains have been realised for TiO_2_ nanotube arrays, which now reach incident-photon-to-current efficiencies approaching 90% in the UV regime [42]. Nevertheless, the increase in the interfacial area must be balanced against a potential rise in surface-state density; thus, nanostructuring is most effective when combined with defect passivation or catalytic overlayers.

### 3.2. Electronic Doping: From Single-Element to Co-Doping

Aliovalent substitution remains the most direct route to modulate the electronic structure of oxide photoanodes. Single-element donors such as W^6+^ → V^5+^ in BiVO_4_ [47], Sn^4+^ → Fe^3+^ in α-Fe_2_O_3_ [45], and Nb^5+^ → Ti^4+^ in TiO_2_ decrease bulk resistivity, steepen band bending, and cathodically shift the photocurrent onset. More recently, co-doping has emerged as a powerful strategy for simultaneously tuning carrier density, defect chemistry, and light absorption. In BiVO_4_, Mo^6+^ or Fe^3+^ donors combined with Sr^2+^ or N^3−^ acceptors increase electron mobility while introducing shallow hole traps that suppress surface recombination; an Fe–N co-doped, nanoporous BiVO_4_ photoanode coated with FeNiOOH currently holds the record photocurrent of ~7 mA cm^−2^ at 1.23 V_RHE [29]. Comparable synergistic effects are observed in α-Fe_2_O_3_ when Sn^4+^ donors are paired with Si^4+^ lattice rigidifiers, yielding ~35% higher photocurrent than Sn-only analogues. Such results highlight three empirical design principles: (i) one dopant should increase majority-carrier concentration, the second should passivate deep traps; (ii) ionic-radius mismatch must remain below ~4% to avoid phase segregation; and (iii) dopant–vacancy binding energies exceeding ~0.5 eV help stabilize defect complexes. Machine learning models that merge ionic descriptors with DFT-derived formation energies now enable predictive ranking of co-dopant pairs prior to synthesis, thereby accelerating experimental exploration [61].

### 3.3. Interface Engineering: Catalysts, Homo-Junctions, and Heterojunctions

Because the semiconductor electrolyte interface is the dominant locus of charge recombination, its rational modification is indispensable for high performance. Ultrathin Co-Pi, Ni(Fe)OOH, and dual FeOOH|NiOOH catalysts dramatically accelerate oxygen evolution, lower surface hole density, and decrease the onset potential of BiVO_4_ by up to 300 mV [46]. Internal homo-junctions created by compositional or phase gradients—such as α|β-Fe_2_O_3_ core–shell nanorods or W-concentration gradients in BiVO_4_—introduce built-in electric fields that halve bulk recombination and lift the applied-bias photon-to-current efficiency to ≈3%. Heterojunctions between distinct semiconductors provide yet another vector for charge-separation engineering. In type-II systems such as BiVO_4_ WO_3_, electrons are preferentially transferred to the lower-lying WO_3_ conduction band, whereas holes remain in BiVO_4_; the resulting bilayer delivers ≈60% higher photocurrent than either constituent alone [59]. Z- or S-scheme architectures (e.g., SnO_2_|BiVO_4_) preserve strong redox potentials by internally recombining low-energy carriers, effecting a ~250 mV cathodic shift in onset potential relative to pristine BiVO_4_. Optimal heterojunctions are characterised by conduction-band offsets of 0.1–0.4 eV, contact resistivities < 1 Ω cm^2^, and parasitic absorption below 10%. Notably, machine-learning-guided screening of more than two hundred oxide pairs predicted BiVO_4_ β-Ga_2_O_3_ and α-Fe_2_O_3_ In_2_O_3_ as promising combinations; both were subsequently validated experimentally, affording 30–45% increases in photocurrent [62].

Collectively, the synergistic application of nanostructuring, advanced (co-)doping, and interface engineering has elevated benchmark one-sun photocurrents to ~7 mA cm^−2^ for BiVO_4_ and ~5 mA cm^−2^ for α-Fe_2_O_3_—values that approach theoretical limits for single absorbers with 2.4 eV and 2.1 eV band gaps, respectively. Yet further progress will depend on data-driven navigation of the multidimensional design space, a topic addressed in the subsequent sections.

## 4. Machine Learning in Materials Discovery for PEC Applications

The application of machine learning to materials discovery—often termed materials informatics—has grown explosively with the availability of large materials databases and advances in algorithms [63,64]. In the context of PEC water splitting, ML can accelerate the identification of promising photoanode compounds by learning from both computational and experimental data. Traditional trial-and-error searches are slow and may miss complex multi-component oxides. By contrast, ML models can sift through vast compositional spaces and pinpoint candidates with desired bandgaps, band edge positions, and stability, even before those materials are synthesized [65,66]. Several key developments underpin this new approach:

### 4.1. High-Throughput Computing and Data Generation

Over the past decade, initiatives like the Materials Project have computed properties (band structures, formation energies, etc.) for tens of thousands of inorganic compounds using DFT [64]. The Materials Project, for example, provides a searchable database of computed bandgaps and stability for known and hypothetical materials [64]. Such repositories enable data-driven screening: one can query for materials that meet specific criteria (e.g., bandgap 1.8–2.8 eV and containing only earth-abundant elements) and obtain a list of candidates for further consideration [64,67]. However, DFT alone has limitations—standard DFT often underestimates semiconductor bandgaps and may not account for surface chemistry or kinetics relevant to PEC performance [68]. To address this, researchers integrate high throughput computing with machine learning corrections and experimental feedback. For instance, Noh et al. employed a hybrid ML/DFT scheme to screen >7000 hypothetical ternary metal oxides for photoanode applications [69]. They trained a crystal graph convolutional neural network (CGCNN) model on a subset of DFT-calculated formation energies to rapidly predict the stability of other compounds, incorporating uncertainty estimates to decide which candidates to validate with additional DFT calculations [69]. This approach significantly reduced the computational cost and identified stable Mg–Mn–O oxides as potential photo absorbers [69]. Similarly, Pilania et al. applied ML to DFT data to predict bandgaps of double perovskites across composition space, revealing several narrow-bandgap oxides that could be useful light absorbers [70]. These examples illustrate how ML can function as a surrogate model for DFT—once trained, the ML model evaluates properties in milliseconds, enabling a virtually exhaustive search of chemical space that would be infeasible with brute-force DFT.

### 4.2. Predicting Material Properties Relevant to PEC

Beyond bandgap and stability, a viable photoanode must have proper band edge alignment with water redox levels and good charge transport properties. These are complex properties not directly tabulated in databases. ML models have been developed to predict such higher-level features by learning from multiple data sources.

**Figure 2:**
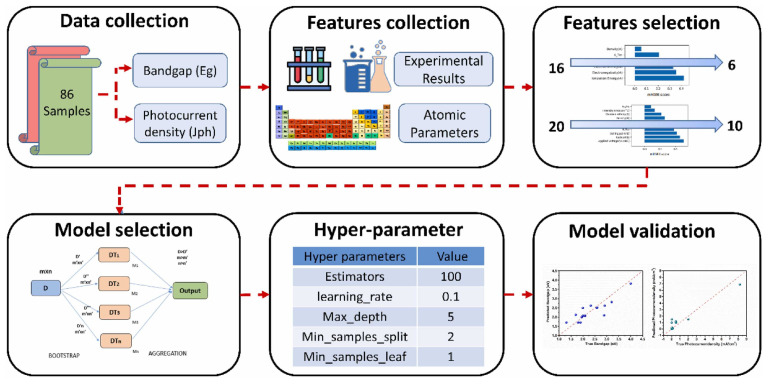
Schematic overview of the machine learning pipeline used for predicting key PEC-relevant properties [71]. Copyright, 2025 Elsevier.

A study by Chen et al. combined DFT-calculated bandgaps, carrier effective masses, and dopant formation energies for a set of oxides with an ML classifier to predict whether a given oxide would exhibit photoanodic activity [35]. The model, though qualitative, correctly recognized known photoanodes and suggested a few novel compounds for experimental testing [35]. Notably, some of the ML-recommended oxides (e.g., a complex perovskite alloy) were later confirmed experimentally to be photoactive. In this work, two machine learning models were constructed to predict the bandgap and photocurrent density of ternary metal oxides (TMOs), and a large number of TMOs were screened based on the models to identify promising candidates for PEC water splitting [71]. Figure 2 illustrates an integrated computational–experimental–machine learning (ML) workflow developed to accelerate the discovery and optimization of nanostructured metal oxide photoanodes for photoelectrochemical (PEC) water splitting. The process includes data collection from 86 samples, feature extraction from atomic parameters and experimental results, feature selection, model selection using decision trees, hyperparameter tuning, and final validation against experimental values of bandgap and photocurrent density.

**Figure 3:**
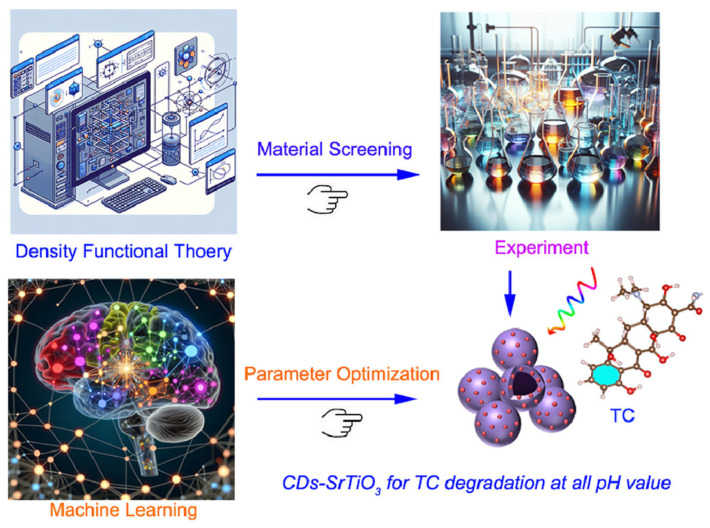
Integrated workflow combining DFT, experiment, and machine learning for photoanode design and optimization [72]. Copyright, 2025 Elsevier.

The process begins with high-throughput density functional theory (DFT) calculations to screen candidate materials based on key physicochemical parameters, such as bandgap, carrier effective mass, and thermodynamic stability. Promising materials identified through theoretical screening are subsequently subjected to experimental validation to evaluate their actual PEC performance under various pH conditions, including photocurrent density and photostability (Figure 3) [72].

Unsupervised ML methods have also been used in materials discovery to organize and interpret large materials datasets. Clustering algorithms applied to computed materials properties can group compounds with similar behavior, potentially highlighting why certain chemistries work well. For instance, unsupervised analysis of a large set of metal oxides based on features like bandgap, electronegativity, and atomic orbitals revealed distinct clusters corresponding to d^0^ and d^1^ transition metal oxides that correlate with good photocatalytic performance [66]. Such analyses help formulate design rules (e.g., presence of d^0^ cations often leads to suitable band edge positions for OER [66]).

### 4.3. Combining Experimental Data and ML

**Figure 4:**
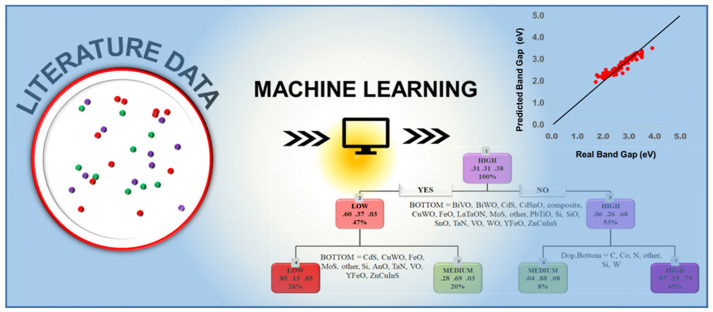
Machine-learning-based band gap prediction and classification of metal oxides [73]. Copyright, 2022 Elsevier.

While computational databases are valuable, they may not capture real-world complexities (surface states, defects, etc.). Therefore, a crucial development is the aggregation of experimental PEC data for ML analysis [23]. Oral et al. recently compiled an extensive dataset of over 10,000 data points from 180 publications on n-type semiconductor photoanodes, including material compositions, nanostructures, and measured photocurrents under standard conditions (Figure 4) [73]. Figure 4 presents a machine learning framework for predicting and classifying the band gap of metal oxides based on literature-derived data. The model is trained on experimentally reported band gap values and successfully captures the structure–property relationships, as demonstrated by the strong correlation between predicted and actual band gaps. The resulting decision tree enables classification of materials into low, medium, and high band gap categories, facilitating efficient pre-screening of potential photoactive candidates.

By applying ML models (random forests and decision trees) to this literature-derived dataset, they could identify which parameters most strongly influence photocurrent. The ML analysis indicated, for example, that the presence of an oxygen evolution catalyst and the use of nanostructured morphology were among the dominant positive factors, whereas too wide a bandgap or insufficient doping density were limiting factors [74]. Such insights, distilled from a massive body of experiments, provide quantitative validation for strategies that researchers intuitively believed important. More impressively, the trained model by Oral et al. was used to predict the outcomes of hypothetical photoanode configurations that had not yet been tried, suggesting specific dopant combinations and catalyst treatments that could yield improved performance [73]. This demonstrates a “knowledge extraction” aspect of ML in materials science: the ability to mine the collective experimental experience and point to fruitful new experiments.

Another example of experimental data-driven discovery is the work by Wu et al. who developed a machine learning model to predict the photocurrent of binary metal oxide photoanodes from synthesis conditions and material properties [75]. They compiled a database of results for various metal oxide photoanodes (BiVO_4_, Fe_2_O_3_, WO_3_, etc.) including film thickness, doping level, annealing temperature, and measured photocurrent. Using an ensemble ML algorithm, they achieved a model that could predict photocurrent within ~10% accuracy for unseen conditions [75]. The model was then employed in an inverse design mode: scanning across possible synthesis parameters to maximize predicted photocurrent for each material. In this way, they identified optimized conditions (e.g., an annealing temperature and film thickness combination) for each oxide that were not obvious from individual experiments [75]. Some of these model-recommended conditions led to higher photocurrents when tested, illustrating how ML can guide optimization even in well-studied materials.

### 4.4. Accelerating Discovery of New Compounds

High-throughput experimental techniques, such as composition spread libraries and parallel synthesis, when paired with ML, form an efficient pipeline for new material discovery. Gutkowski and coworkers pioneered this approach by combining automated experimentation with data analytics to find new photoanodes [76]. In one study, they fabricated a gradient composition library of Bi–V–Mo oxides and used scanning PEC microscopy to map photoactivity. ML clustering of the activity data revealed a compositional sweet spot corresponding to BiVO_4_ doped with a few percent Mo, which aligned with the known optimal BiVO_4_: Mo composition—effectively rediscovering it in a rapid manner and providing proof of concept. Looking forward, active learning algorithms can be used to drive such experiments: the ML model analyzes initial screening results and decides on the next set of compositions to test for maximizing information gain or performance [65,66]. Montoya et al. demonstrated an active learning approach for electrocatalyst discovery, which in principle can be applied to photoelectrode discovery [69]. In their case, an ML model was iteratively retrained on DFT adsorption energy data and used to propose new alloy compositions to evaluate, quickly honing in on promising catalysts. For photoanode materials, a similar active learning loop could drastically reduce the number of experiments needed to pinpoint an optimal multinary oxide or oxynitride.

The integration of ML with quantum chemistry is also opening new frontiers [77]. For example, a recent small-data ML approach was used to efficiently explore Zn–Ti–O high-entropy alloys for photocatalysis, identifying compositions with improved activity by training on as few as a dozen initial samples [66]. While this example was for photocatalytic powder suspensions, the methodology of small-data ML—using physical descriptors and uncertainty-guided sampling—is highly relevant for discovering complex oxide photoanodes where generating large datasets is costly or slow.

In summary, materials discovery for PEC photoanodes has been greatly empowered by machine learning techniques. By leveraging both computational data (for scale and breadth) and experimental data (for ground truth on performance), ML models can uncover candidates and trends far more efficiently than human intuition or brute-force methods. The outcome is a more directed search: rather than synthesizing hundreds of random oxides, researchers can focus on a short list of ML-recommended compounds that satisfy multiple design criteria (bandgap, band alignment, stability) [70]. Indeed, some of the highest performing recently reported photoanodes were first suggested by computational screening or ML analysis before being realized in the lab [29]. As the community continues to accumulate data (e.g., via shared databases of PEC measurements) and improve algorithms, one can envision a “virtual screening” of the periodic table that rapidly yields the next generation of superior photoanode materials. The next section will delve into how machine learning is also being applied to optimize known materials—fine-tuning doping, morphology, and interfaces—which is a complementary effort to discovering new compositions.

### 4.5. Classification of ML Approaches in PEC Applications

To provide a structured overview of the diverse machine learning techniques applied in PEC photoanode research, we propose a taxonomy that categorizes ML methods by both their learning paradigm and specific functional roles within the PEC workflow. Table 1 summarizes the most representative ML models—spanning supervised, unsupervised, Bayesian, and physics-informed learning—and maps them to key application stages in photoanode design and optimization.

This framework not only highlights the versatility of ML in addressing distinct challenges across the PEC pipeline—from initial material screening to device-level integration—but also helps guide future researchers in selecting suitable models based on task-specific needs. Future work could further refine this classification by incorporating model interpretability, data requirements, and computational efficiency.

## 5. ML-Guided Optimization of Material Properties (Doping, Morphology, Interfaces)

Even after a promising semiconductor is identified, achieving its best possible performance often requires navigating a large parameter space of compositional and structural modifications. Machine learning offers powerful approaches to optimize material properties by learning the complex, and sometimes nonlinear, relationships between processing parameters, material structure, and PEC performance. In this section, we highlight how ML techniques have been used to guide doping selection, morphology control, and interface engineering for metal oxide photoanodes [29,78].

### 5.1. Dopant Selection and Composition Optimization

Selecting the right dopant (or combination of dopants) and concentration can make the difference between a mediocre photoanode and a record-setting one [71,79]. Traditionally, dopant exploration was achieved by educated guesses or trial and error. ML can systematize this process [80]. A striking example is the work of Wang et al. [29], who developed a machine learning model to predict the effects of various metal dopants on Fe_2_O_3_ photoanodes. They compiled data on 17 different dopants (from across the periodic table) incorporated into hematite, including features like dopant ionic radius, valence, formation energy, etc., and the resulting photocurrent enhancements from experiments. By training a random forest regression model on this dataset, they achieved accurate predictions of photocurrent for dopant types and levels not yet tested experimentally. The model suggested, for instance, that certain underexplored dopants (like small amounts of Al^3+^ or Ga^3+^) could improve hematite similarly to the well-known Sn^4+^ and Ti^4+^ dopants. It also identified diminishing returns beyond an optimal dopant concentration, guiding researchers on how much doping is beneficial [81]. This ML-guided approach was then extended to another material (CuO) by transferring the knowledge learned on hematite, illustrating how a model trained on one photoanode can inform dopant selection in another oxide with analogous properties [29]. Such cross-learning significantly accelerates the tuning of new materials, sparing researchers exhaustive one-by-one dopant trials [10].

**Figure 5:**
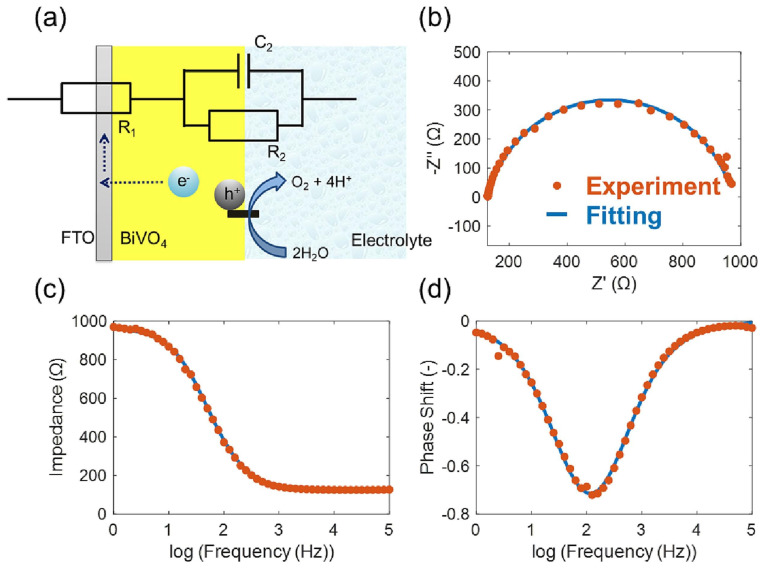
Machine-learning-guided the representative PEIS data and corresponding fitting for a BiVO_4_ photoanode. (**a**) Equivalent circuit model used for fitting. (**b**) Nyquist plot, (**c**) Bode impedance plot, and (**d**) Bode phase plot. Blue lines indicate the fitted curves [85]. Copyright, 2023 Elsevier.

While single-element doping has shown clear benefits [82], recent studies are increasingly focusing on multi-element co-doping strategies, guided by ML optimization algorithms [83]. Beyond single dopants, ML is being used to tackle co-doping and alloy composition optimization—tasks where the parameter space (multiple elements with continuous concentration ranges) becomes very large. A recent study applied Bayesian optimization (an ML strategy for global optimization) to determine the optimal co-dopant concentrations in BiVO_4_ [84]. Starting with a small set of experiments on BiVO_4_ co-doped with Mo and another metal (X = Ca, Sr, or Ba), the authors trained a Gaussian process model to fit the photocurrent as a function of Mo and X doping levels. The Bayesian optimizer then suggested the next best doping ratios to try, targeting maximal photocurrent. Through only a few iterative rounds (a total of ~12 compositions tested), the ML-guided search found an optimal (Mo + Sr) co-doping that outperformed all initial samples, improving photocurrent by ~15% [84]. ML can likewise extract key descriptors from electrochemical-impedance data. Kobayashi et al. modeled BiVO_4_ PEIS with an R_1_∥(R_2_–CPE) circuit (Figure 5a–d); the single semicircle in the Nyquist plot confirmed negligible bulk resistance, and the excellent overlap between experimental and fitted Bode curves validated the model. When the fitted parameters were fed into a Gaussian-process regression, the interfacial pseudo-capacitance C_2_ emerged as the most predictive descriptor, indicating that lowering trap-state capacitance speeds hole transfer and raises the photocurrent [85]. Thus, beyond compositional-space optimization, coupling ML-driven spectral–performance correlation analysis provides an additional, effective avenue for advancing the design of BiVO_4_—and other PEC electrodes.

In compositionally complex oxides, where there may be 3 or 4 cation elements, ML models (particularly those using feature selection techniques) can also help identify which element predominantly controls a certain performance aspect. Kobayashi et al. applied lasso regression (which performs feature selection) to a dataset of BiVO_4_ photoanodes with varying dopants and noticed that the presence of Mo doping emerged as the most significant positive predictor for photocurrent, more so than other doping or processing features [47]. This quantitatively confirmed Mo’s crucial role in BiVO_4_’s performance, aligning with chemical intuition but now supported by data-driven evidence [47]. In cases where chemical intuition is less clear (e.g., co-doping with elements that have opposing effects on carrier density and mobility), such ML analyses can provide guidance on whether the net effect is likely beneficial or detrimental.

### 5.2. Morphology and Microstructure Optimization

The nanostructure of a photo-anode—grain size, porosity, layer thickness and facet exposure—strongly dictates its efficiency. Yet, correlating morphology with performance is difficult because morphology is typically described by many (often qualitative) parameters. Image-based machine learning can overcome this barrier. A pioneering study by Hayashi et al. trained a convolutional neural network (CNN) that converts SEM images of BiVO_4_ films directly into full J–V curves, accurately distinguishing densely sintered, low-performance films from porous nano-architectures with high photocurrent and low onset potential [67]. Interpreting the activation maps revealed that continuous pore pathways and rough surfaces are key morphological motifs, providing actionable guidance for fabrication.

**Figure 6:**
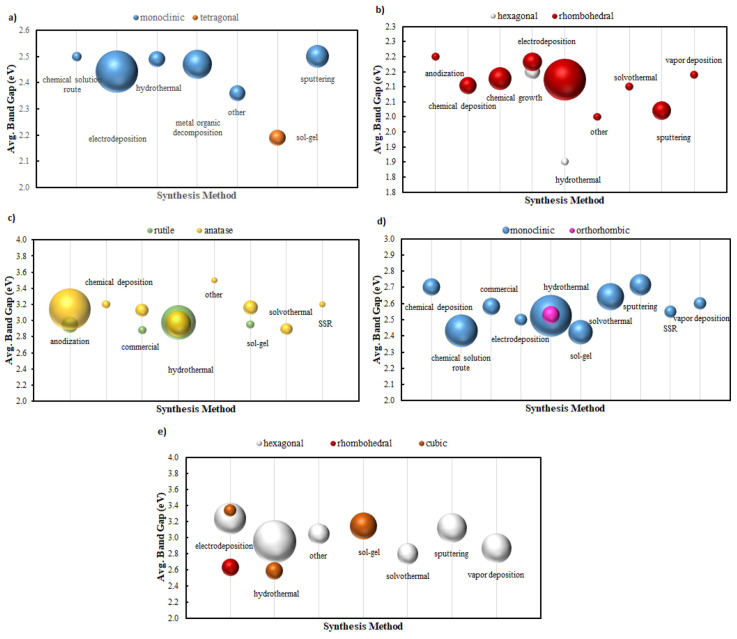
Demonstrates how various synthesis methods influence the crystal structure and band gap energies of five representative metal oxide photoanodes: (**a**) BiVO_4_, (**b**) Fe_2_O_3_, (**c**) TiO_2_, (**d**) WO_3_, and (**e**) ZnO [73]. Copyright, 2022 Elsevier.

Beyond deep-image learning, simpler ML models can optimize numerical descriptors obtained from conventional characterization [86]. For example, Okunaka et al. systematically varied calcination temperature to generate nanoporous BiVO_4_ films and showed, with decision-tree regression, that intermediate firing yields the best compromise between crystallinity and surface area [87]. Facet-controlled BiVO_4_ nanorod arrays prepared by Xiao et al. further underline how preferential exposure lowers electron–hole recombination, an insight that can be captured by feature-selection algorithms [88]. As Figure 6 makes clear, the electronic structure of metal-oxide photo-anodes depends strongly on the chosen synthesis route and resulting crystal phase, emphasizing why these processing descriptors must be supplied to any machine learning model that aims to predict PEC performance.

The efficiency of oxide photo-anodes hinges on nanoscale attributes—grain size, porosity, film thickness and exposed facets—yet converting such rich morphology into quantitative performance rules is non-trivial. Image-based machine learning is beginning to bridge this gap. Hayashi et al. trained a convolutional neural network (CNN) on SEM micrographs of BiVO_4_ films tagged with their measured J–V curves; the model reconstructed the entire photocurrent–voltage profile from a single image with R^2^ ≈ 0.99, reliably flagging porous nano-architectures as high-performing and dense films as poor performers [67]. Saliency-map analysis revealed that continuous pore networks and surface roughness dominated the CNN’s attention, providing data-driven design cues. Such models can also run in inverse-design mode: synthetic microstructures are generated, screened in silico and only the most promising geometries fabricated, enabling closed-loop optimization impossible with conventional analytic models. Similar image-to-property pipelines have already proven generalizable in other functional oxides, e.g., predicting ionic conductivity from micrographs of ceramics [89]—highlighting the broad potential of ML-guided morphology engineering for photoelectrochemical (PEC) devices.

Figure 6 illustrates how different synthesis techniques—such as sol–gel deposition, hydrothermal growth, and spray pyrolysis—affect the measured optical band gaps of representative photoanode materials including BiVO_4_, Fe_2_O_3_, and WO_3_. The observed variation underscores the role of synthetic conditions in tuning material electronic structure, likely due to differences in crystallinity, defect density, and grain boundary effects. Such sensitivity highlights the need to consider synthesis descriptors as key input features in ML models for accurate property prediction and transferability across experimental datasets [51].

Even without advanced image analysis, simpler ML models have been used to optimize morphological parameters obtained from characterization. For instance, one group varied the annealing temperature and film thickness of WO_3_ photoanodes and recorded resulting grain sizes, roughness factors, and photocurrents. They trained a decision tree model on these data, which revealed that an intermediate annealing temperature giving ~50 nm grains maximized photocurrent, whereas temperatures that were too low (amorphous) or too high (excessively large grains, low surface area) were suboptimal [75]. The model also indicated diminishing returns for thickness beyond a certain point (due to light absorption vs. charge transport tradeoff), helping pinpoint an optimal film thickness ~300 nm for their WO_3_ films. These findings guided the fabrication of a WO_3_ photoanode with ~15% higher efficiency than any in the initial dataset, validating the utility of ML in tuning morphological parameters.

### 5.3. Interface and Surface Optimization

ML’s role in interface engineering is emerging. One challenge is that interfaces (e.g., catalyst/semiconductor junctions) are complex and not easily parameterized. Nonetheless, researchers have started to apply ML to decide on catalysts, protective layers, or treatment methods for photoanodes [90].

**Figure 7:**
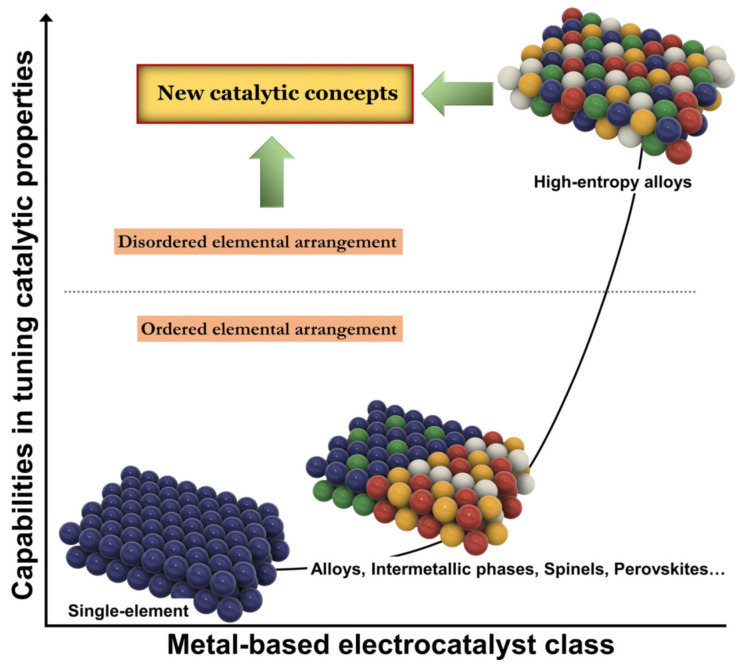
Catalyst design space evolution from single-element to high-entropy alloys [96]. Copyright, 2021 Angewandte Chemie International Edition.

Machine learning tools are now being applied to the less-tangible—but performance-critical—interface between the light absorber and its catalytic or protective overlayer. Liu et al. showed that surface-fluorinating BiVO_4_ introduces upward band-bending and suppresses electron traps, shifting the onset potential by ≈0.3 V and prolonging stability [91]. Using a dataset of diverse post-treatments, Chen et al. trained a clustering/classifier model that flags surface modifications likely to yield such favorable band-bending from quick impedance-spectroscopy read-outs [92]. On the catalyst side, Moon et al. employed small-data active learning to locate an optimal four-metal perovskite OER catalyst in <20 experiments [93], while Ding et al. combined data mining, active learning and domain adaptation to down-select acidic Ru-Mn-Ca-Pr oxides from thousands of possibilities [94]. A human–machine closed loop then enabled Sakaushi et al. to traverse ≈3000 quinary Ni-Fe-Mn-Co systems and deliver a PGM-free catalyst rivaling RuO_2_ in only two iterations [95]. Figure 7 visualizes how increasing the structural and elemental complexity of metal-based electrocatalysts—from ordered alloys to disordered high-entropy alloys (HEAs)—can vastly broaden the design space for interfacial tuning. Such compositional flexibility is especially well-suited to machine learning strategies, which can efficiently explore and optimise multivariate catalyst systems for photoelectrochemical (PEC) applications [96,97]. Moreover, high-entropy alloys and oxides at the photoanode–catalyst interface exploit configurational entropy and multi-element synergy to finely tailor adsorption energies and lattice distortions, resulting in superior OER kinetics and enhanced interfacial stability [98].

### 5.4. Process Optimization and Upscaling

Machine learning is no longer confined to composition and morphology; it is now optimising the processing knobs that ultimately determine scalability. Sahu et al. trained an artificial-neural-network surrogate on 297 BiVO_4_ electrodeposition runs spanning bath pH, current density and charge passed; an in silico sweep of >10,000 virtual trials singled out a pH-6.2 and 0.18 C recipe that was later verified to boost photocurrent by ~20% [99]. Using Bayesian hyper-parameter tuning, Wahab et al. refined a two-step annealing ramp for graphene-TiO_2_ thin films, achieving densification without grain coarsening and a 30% rise in PEC activity [100].

Once a surrogate is in hand, feature-importance analysis can reveal which levers genuinely matter: Kobayashi et al. showed that W-doping charge and catalyst coverage, rather than film thickness, dominate performance variance in BiVO_4_ photo-anodes [85]. These closed-loop ideas are now being embedded in automated laboratories; Kormányos, Jenewein and Cherevko describe a “plan-synthesise-measure-learn” robot that couples high-throughput deposition with Gaussian-process optimisation [101], while Pence et al. demonstrate a Bayesian algorithm that live-tunes electrodeposition waveforms, cutting optimisation time from weeks to hours [102].

**Figure 8:**
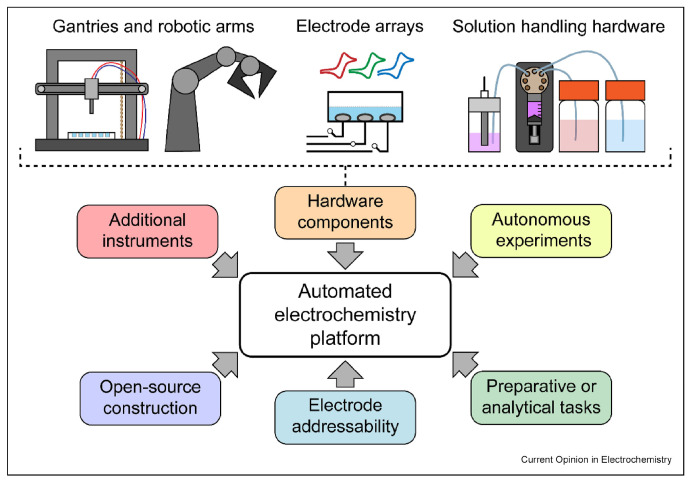
Automated electrochemistry platforms for ML-integrated experimentation [102]. Copyright, 2025 Elsevier.

In practice, ML-guided optimization has led to notable successes. For BiVO_4_, as discussed, ML pinpointed co-doping and catalyst schemes that achieved record performance. Figure 8 depicts the modular architecture of automated electrochemistry platforms like PANDA 2.2.2 under Python 3.10.12 [102], where robotic systems and addressable electrodes enable ML-guided, closed-loop PEC material screening. For hematite, ML recommended unconventional dopants that experimentally yielded comparable improvements to well-known ones [29]. For complex oxides like triple-cation oxides, ML has managed to maintain performance while reducing expensive components, by navigating trade-offs. These achievements underscore that ML is not just a theoretical exercise—it directly produces better materials. There is still room to grow: many studies so far use relatively small or domain-specific datasets. As community-wide data sharing increases, one can envision more universal models that can predict, for example, the needed dopant and structure for any given oxide to reach a target performance. Eventually, ML-optimized materials will feed back into ML-driven discovery (and vice versa), closing the loop between discovering new materials and fine-tuning them to their full potential.

### 5.5. Considerations in Model Selection, Interpretability, and Uncertainty Quantification

Effective machine learning in PEC photoanode research requires not only high predictive accuracy but also model transparency and reliability. While deep learning models such as CNNs excel at tasks like morphology-to-J–V prediction, they often function as “black boxes” and may overfit or misinterpret noise—especially when trained on limited or unbalanced data [67].

In contrast, tree-based models (e.g., random forests) offer better interpretability and have successfully identified key parameters like dopant electronegativity or catalyst coverage [17,51]. Tools such as SHAP and LIME are increasingly used to explain model outputs, helping researchers understand which features drive performance trends. For example, SHAP analysis has shown that W-doping and surface catalyst loading strongly influence BiVO_4_ performance, even more than film thickness [85].

Uncertainty quantification—via Bayesian methods or dropout-based neural nets—adds an additional layer of model trustworthiness. It allows researchers to identify low-confidence predictions and prioritize them for further validation. As the field advances, integrating explainability and uncertainty into ML workflows will be essential for producing not just accurate, but also scientifically meaningful, predictions.

## 6. Performance Prediction and Device-Level Integration

Machine learning is not only accelerating materials discovery and optimization at the material level but is also increasingly employed to predict the performance of complete photoelectrochemical systems and to assist in the integration of photoanodes into devices. In this section, we examine how ML models are used to predict key performance metrics (like J–V curves, incident photon conversion efficiency, stability) and how they facilitate the design of integrated PEC devices (such as tandem cells or wireless water-splitting units). The ability to rapidly predict device-level outcomes is crucial for evaluating design choices without always resorting to time-consuming experiments or complex physics-based simulations.

### 6.1. Comparative Evaluation of ML Models in PEC Applications

While numerous machine learning studies have reported success in predicting PEC-relevant properties, their comparative performance, generalizability, and data dependency are rarely benchmarked in a unified manner. Table 2 summarizes representative ML models used for key PEC tasks, along with their input features, dataset scale, target outputs, validation methods, and quantitative performance indicators. This comparison provides a clearer picture of the strengths and limitations of each approach.

Among these, CNN-based models excel at capturing morphological nuances directly from imaging data, while ensemble models like random forests offer better interpretability and robustness when trained on structured features. However, performance consistency is heavily dependent on data quality and diversity. Notably, models trained on synthesized datasets (e.g., Oral et al.) generalize poorly to novel chemistries, highlighting the need for cross-validation across material classes. Future work should focus on developing benchmark datasets and standard performance metrics to enable more transparent model comparison.

### 6.2. Predicting Photocurrent–Voltage Behavior

Machine learning (ML) surrogates have progressed from single-point regressors to fast, physics-aware digital twins able to reproduce an entire J–V response in a few milliseconds. A landmark demonstration by Hayashi et al. trained a convolutional neural network (CNN) on paired SEM micrographs and experimental J–V curves for BiVO_4_ films; the network delivered a mean coefficient of determination R^2^ = 0.95 across an external test set, cleanly separating dense, low-performing electrodes from porous, high-current architectures [67]. Because the predictor accepts raw images, it bypasses manual descriptor engineering and can be embedded directly in an automated microscope-to-model workflow.

Image-free surrogates offer complementary insight. Sahu et al. encoded 33 descriptors—intrinsic electronic properties, bath pH, deposition charge, and NiOOH cocatalyst presence—for 297 electrode-positioned BiVO_4_ samples and trained an artificial neural network (ANN) that reproduces the photocurrent at eleven bias points with a mean absolute error of 0.03 mA cm^−2^; in silico screening of 10,000 virtual recipes singled out a pH 6.2 and 0.18 C condition that raised the real-world photocurrent by 20% [99]. Shapley additive explanations (SHAP) ranked donor density and NiOOH coverage as the two dominant levers, providing a quantitative counterpart to classical semiconductor theory.

Feature-importance studies extend beyond BiVO_4_. Kobayashi et al. combined random-forest regression with SHAP to analyse a library of 136 BiVO_4_ photo-anodes synthesised under varied thicknesses, dopant doses and catalyst loadings; the model confirmed that film thickness offers diminishing returns above ~400 nm because increased absorption is offset by charge-collection losses [85].

The approach generalises to other oxides. Diaby et al. developed an ANN that maps deposition time, annealing temperature and electrolyte pH to the PEC behaviour of WO_3_/steel electrodes with <0.05% relative error; wrapped inside a Bayesian optimiser, the surrogate cut the experimental search space by an order of magnitude [103].

In parallel, physics-informed networks are emerging. Ren et al. embedded drift–diffusion constraints into a Bayesian neural network for GaAs photoelectrodes, retaining interpretability while achieving a 100-fold speed-up over finite-element solvers [104]. Another compelling study by Diaby et al. leveraged an ANN to predict the PEC behavior of WO_3_ photoanodes from fabrication parameters [103]. By training on data from WO_3_ films grown under various conditions, their model achieved <0.05% error in photocurrent prediction, essentially functioning as a virtual WO_3_ photoanode experiment. When integrated into a feedback loop, this allowed rapid identification of deposition conditions that maximize photocurrent.

Taken together, these studies illustrate how ML shrinks the feedback loop from hours of characterization or minutes of numerical simulation to seconds of prediction, enabling rapid “what-if” interrogation of material and processing variables before any wafer-scale fabrication is attempted.

### 6.3. Device-Level Integration—Tandems and Modules

Classical optical and drift-diffusion simulations explore only a fraction of the multi–parameter design space (band-gap pair, individual layer thicknesses, antireflection coatings, catalyst absorption, etc.). Yi et al. replaced time-consuming finite-difference time-domain (FDTD) sweeps with a deep neural network trained on 8000 simulated perovskite/Si tandem structures; the surrogate predicted the cell photocurrent density with 98% accuracy and, when coupled to a genetic algorithm, identified an optical stack that improved the projected efficiency by 28% in milliseconds rather than days [105].

For genuine PEC tandems, both optical and electrochemical constraints must be respected. Vilanova et al. constructed a BiVO_4_∥c-Si tandem (“CoolPEC”) and showed, through coupled optical/electrical modelling, that antireflection-optimised BiVO_4_ (550 nm) atop a 2 µm Si bottom cell can in principle exceed 5% STH; their experimentally validated design achieved 2.3% on a 50 cm^2^ module [106]. A complementary computational screening by Seger et al. used high-throughput density-functional datasets to confirm that the 1.7 eV/1.0 eV band-gap combination is indeed the global optimum when catalyst optical losses are included [107].

Going beyond forward prediction, surrogate models are now embedded in Bayesian or reinforcement-learning loops that iterate towards Pareto fronts of efficiency, stability and cost. Oviedo et al. integrated an auto-encoder device surrogate with Bayesian active learning to co-optimize layer thicknesses and interfacial recombination parameters in hybrid PEC–PV tandems, pruning a 100,000-point search space to <200 physical experiments while reaching the theoretical current-matching limit.

One approach is to use ML surrogate models in conjunction with optical/electrical simulators to perform tandem device optimization. Hu et al. had analytically determined that an ideal tandem for water splitting consists of a ~2.0 eV top absorber and ~1.1 eV bottom absorber to maximize efficiency [108]. ML can refine this by incorporating more details: for example, accounting for realistic absorption spectra and non-ideal charge transport. An ML model (like a neural network) can be trained on a dataset of simulated tandem device performances (where each data point is a specific combination of top cell thickness, bottom cell thickness, bandgaps, etc., and the output is the predicted STH efficiency) [108]. Once trained, the ML model can very quickly explore the parameter space and find the optimum, which might be something like “a 550 nm BiVO_4_ top cell with a 1.8 eV bandgap and a 2 µm CIGS bottom cell with 1.0 eV bandgap yields 5% STH” [108].

### 6.4. Stability and Lifetime Predictions

Another critical aspect is predicting durability of photoanodes, as stable operation over thousands of hours is required for practical deployment. ML is being leveraged to predict degradation behavior by learning from accelerated aging tests. For instance, by monitoring features such as photocurrent decay rate, spectral response changes, and electrochemical impedance over many stress tests, one can train an ML model to predict the photoanode’s lifetime (time to 20% drop in performance) from initial diagnostic data. Mastronardi et al. applied such an approach to TiO_2_-protected III-V photoelectrodes: an ML model was trained on initial electrochemical impedance spectra and could predict whether the device would fail (due to pinhole formation in TiO_2_) within a given time window [109]. The model identified certain impedance signatures that correlate with eventual failure, enabling a kind of “early warning.” Translating that to metal oxide photoanodes, similar techniques could predict, for example, the onset of photocorrosion or catalyst delamination based on initial transients or voltametric fingerprints. While not yet widespread, this area is growing, as seen by efforts to use probabilistic ML models to estimate the distribution of possible degradation rates under different conditions [109]. Such predictions are invaluable for designing robust devices—one can virtually screen which protection layer (or which electrolyte pH, etc.) will give an acceptably long life.

### 6.5. System-Level Optimization and Control

Beyond the device itself, ML can optimize the operation of PEC systems. For example, in a solar-hydrogen generator that uses a concentrating lens and a PEC cell array, an ML controller could adjust the angle or intensity distribution to maximize hydrogen output while avoiding hotspots. There are demonstrations of ML-based controllers in photovoltaic fields and in electrolyzes; applying similar control logic to PEC devices (e.g., adjusting bias or light splitting dynamically to respond to clouds) can improve efficiency and durability. Counterintuitively, they found that a short, pulsed illumination with certain duty cycle allowed time for slow OER intermediates to convert, boosting net oxygen yield—something a straightforward test might not have revealed. This indicates ML can help devise operational strategies (like pulsed illumination or periodic biasing) to eke out better performance from existing materials.

On the manufacturing side, if PEC cells are to be scaled up into modules, ML is useful for quality control and uniformity optimization. Imaging-based ML can quickly flag defects or non-uniform regions in large-area photoanodes that could lead to failure. Predictive maintenance of a PEC array might also rely on ML algorithms analyzing performance data to schedule cleaning or replacement.

In summary, ML at the device and system level ties together the advances made at the materials level. It enables rapid what-if analysis for new device architectures (tandems, multi-junctions, wired vs. wireless configurations), it helps ensure that improvements at the material level indeed translate to higher STH efficiencies, and it provides tools to address practical issues like stability and operational control. As an illustration, many of the recent record solar-to-hydrogen efficiencies have come not just from a single breakthrough material, but from careful integration of a photoanode with other components [68]. ML can streamline that integration: for instance, by predicting how a change in the photoanode will necessitate a change in the photocathode or the bias conditions to maintain optimal current matching. With ML able to juggle these multivariate dependencies, designing a fully integrated PEC system becomes a tractable optimization problem rather than a daunting multidimensional trial.

### 6.6. Example—Tandem Design Case Study

Consider designing a tandem PEC device using a BiVO_4_ photoanode and a silicon photovoltaic cell in a bias-free configuration. Without ML, one might experimentally vary BiVO_4_ thickness, doping, and the area ratio between BiVO_4_ and Si, etc., in a sequential manner. An ML approach, by contrast, could employ a model trained on a small number of measured configurations to predict the photocurrent and operating voltage of the tandem for untried configurations. Say the initial data suggests that increasing BiVO_4_ thickness boosts light absorption but hurts charge collection beyond 300 nm, and that the Si cell can provide ample current (~35 mA cm^−2^) but the two must be current-matched. The ML model might then predict an optimal BiVO_4_ thickness of ~250 nm (slightly thinner to avoid excess absorption beyond BiVO_4_’s charge collection depth) and recommend lightly Mo-doping BiVO_4_ to raise its photocurrent to ~7 mA cm^−2^ while using a tandem area ratio such that the Si cell current is also ~7 mA cm^−2^ at the operating point [68]. It might also suggest adding a thin TiO_2_ interlayer to reduce interface recombination if that was part of the input features it learned from. Acting on these predictions, one could rapidly assemble a tandem that operates near the sweet spot, whereas a manual search might have missed the precise combination of thickness and doping that yields current matching. In fact, Jia et al. achieved a bias-free water splitting efficiency of ~2.5% using a tandem of Mo:BiVO_4_ and c-Si bottom cell, guided in part by optical-electrical modeling that is conducive to ML acceleration [68]. As ML models grow to incorporate both optical and electrochemical phenomena, they will become indispensable for such device engineering.

Finally, it is worth noting that device-level ML does not operate in isolation—it benefits immensely from the material-level ML discussed earlier. The better the photoanode materials become (often thanks to ML-guided optimization), the easier the job of device integration. Conversely, device-level requirements can feed back to materials discovery. For instance, a device simulation might reveal that a photoanode with slightly wider bandgap but higher photovoltage yields better tandem efficiency than one with maximum photocurrent but lower voltage. That insight could direct discovery efforts (via ML) to prioritize materials with those characteristics. Thus, an end-to-end ML-augmented design loop emerges from atoms (material composition) to device (system efficiency) and back.

### 6.7. Experimental Integration and Feedback-Driven Model Refinement

The integration of machine learning with experimental workflows is essential for translating computational predictions into tangible advances in PEC photoanode development. Although recent studies have demonstrated the predictive power of ML in identifying promising dopants, morphologies, and synthesis parameters, a clear methodology for experimental validation remains underdeveloped. To enable effective implementation, researchers should couple ML-generated hypotheses with standardized synthesis and PEC characterization protocols. For example, candidate materials such as BiVO_4_ co-doped with Sr and Mo, identified through data-driven optimization, can be fabricated via conventional thin-film deposition techniques and evaluated under uniform testing conditions, such as AM 1.5 G illumination and pH-controlled electrolytes. This ensures the comparability and reproducibility of experimental results across different research groups.

Importantly, the results of such experiments should not remain isolated, but instead be reintegrated into the ML model to refine its predictive accuracy. Deviations between predicted and observed performance—such as discrepancies in photocurrent density or onset potential—can reveal limitations in the original feature set, model architecture, or training data diversity. Incorporating this feedback enables the construction of closed loop learning frameworks, where the model continuously improves by learning from new experimental data. This iterative cycle of prediction, validation, and refinement represents a critical step toward autonomous materials discovery and will be central to the future development of scalable, high-performance PEC systems.

## 7. Challenges and Future Outlook

Despite the demonstrated promise of machine learning in accelerating PEC photoanode development, several critical challenges remain. Chief among these is the scarcity and heterogeneity of high-quality data. Experimental PEC datasets are often limited in size, lack standardization in measurement conditions, and are biased toward positive results. To address this, the development of community-wide benchmark datasets—featuring standardized J–V curves, illumination protocols, and electrolyte environments—is essential. Additionally, synthetic data augmentation using physics-based simulations, such as density functional theory (DFT) or drift–diffusion models, can expand the training space and provide physically meaningful labels where experiments are impractical or costly.

Another persistent issue is model generalization and interpretability, especially when training data are sparse or domain-specific. Transfer learning offers a viable solution by allowing models trained on larger, related datasets (e.g., bulk oxide properties or electrocatalytic performance) to be adapted to PEC-specific tasks with minimal retraining. Moreover, incorporating physical constraints into model architectures, or using hybrid ML-physics models, can prevent unphysical predictions and improve extrapolation reliability. Ultimately, combining synthetic data, transferable models, and standardized reporting practices will be key to overcoming current limitations and establishing a robust, generalizable ML framework for PEC materials discovery.

The successful examples surveyed in this review make a compelling case for the power of machine learning in advancing PEC photoanodes. However, realizing the full potential of ML-guided design in this field requires overcoming several challenges. In this section, we discuss key hurdles—including data limitations, model interpretability, and experimental integration—and outline future directions that could address these issues, paving the way for a new paradigm of photoanode development.

### 7.1. Data Quality and Availability

A fundamental challenge is the scarcity of high-quality data for training robust ML models [110]. Developing a predictive model requires consistent, relevant data—yet in academic literature, PEC performance results are often reported under varying conditions (different light sources, electrolytes, cell configurations). This heterogeneity complicates the direct use of published data for ML. Moreover, negative or inconclusive results are seldom published, leading to publication bias where models are trained only on successful experiments, potentially skewing their predictions. To improve this situation, the community is moving toward more standardized reporting (e.g., using standard AM 1.5 G illumination and reporting photocurrent at 1.23 V_RHE) and data sharing. Recent efforts like the PEC data platform encourage researchers to upload raw J–V curves, IPCE spectra, and stability data in a common format. As these databases grow, ML models will benefit from larger and more diverse training sets, improving their accuracy and generalizability. Collaborative benchmarking exercises—akin to the “blind challenges” in the computational chemistry community—could be initiated, where multiple groups contribute data on, say, BiVO_4_ photoanodes under a fixed protocol. An ML model can then be trained on part of this dataset and tested on the rest to objectively evaluate its predictive power. Overcoming data scarcity may also involve synthetic data generation: physics-based simulators (validated on smaller datasets) can produce additional training data for ML, a strategy known as transfer learning when moving between simulated and real data domains.

### 7.2. Feature Selection and Interpretability

Many ML models, especially deep learning ones, are often criticized as “black boxes” that provide predictions without clear explanations [63]. In materials science, understanding why a certain dopant is effective or how a nanostructure improves performance is as important as the prediction itself. There is a strong push for explainable AI (XAI) in this field. Methods like SHapley Additive exPlanations (SHAP) and Local Interpretable Model-Agnostic Explanations (LIME) can be used to interpret complex models by attributing importance to input features. In one case, an ML model trained on mixed-metal oxide photoanodes indicated that the electronegativity difference between constituent elements was a critical descriptor for high activity—a somewhat surprising insight that hints at the role of polar covalent bonding in facilitating charge transfer. Such interpretable outcomes are extremely valuable as they can inspire new hypotheses and deepen scientific understanding beyond the raw data. The development of materials-specific XAI tools is an active area of research [65]. Future ML studies on photoanodes will likely incorporate partial dependence plots, feature importance rankings, and even symbolic regression (which fits human-readable formulas) to ensure that models do more than predict—they also elucidate. As an illustrative vision, one might imagine an ML model that not only tells us “doping with element X at Y% will improve the photocurrent by 20%” but also provides an explanation—“because element X raises the Fermi level and improves band bending, leading to a 15% increase in charge separation efficiency”—bridging the gap between data-driven prediction and mechanistic theory.

### 7.3. Generalization and Extrapolation

Most ML models perform interpolation—they make reliable predictions within the range of data they were trained on, but can fail when asked to extrapolate beyond that range. In the context of materials, a model trained on certain compositions might not predict well for a radically different composition outside its experience. This raises a concern: if we only train on known materials, will ML ever propose truly novel materials? One way to encourage novelty is through active learning where the model identifies regions of high uncertainty (often at the edges of the current data distribution) and suggests experiments there. This way, the model’s domain of knowledge expands iteratively. Another approach is to incorporate physical constraints into ML models—for instance, enforcing that predicted efficiencies cannot exceed the thermodynamic limit, or that certain trends (like the effect of bandgap on achievable photocurrent) follow known physics (the Shockley–Queisser type limit). So-called physics-informed machine learning combines data-driven flexibility with hard-wired physical laws to improve extrapolation trustworthiness. For PEC systems, one could embed the Butler–Volmer kinetics or space-charge region theory into a loss function that the ML model optimizes, thereby constraining its behavior at extremes. The future likely holds hybrid modeling approaches where ML handles the complex, unknown parts of the system (e.g., defect chemistry impact on performance) while analytic physics handles the well-known parts (e.g., bandgap vs. maximum photocurrent). This will not only improve generalization but also reduce the amount of data needed for training.

### 7.4. Integration with Experiment (Closing the Loop)

While ML models can make predictions, realizing their benefits depends on executing the recommended experiments and feeding the results back—essentially creating a closed-loop autonomous discovery system. Realizing this vision poses practical challenges: experiments on photoanodes can be time-consuming (synthesis plus testing might take days per sample) and not easily automatable compared to, say, synthesizing molecules in a flow reactor. However, progress is being made in automation: high-throughput inkjet printing or sputtering systems can create compositional gradients of oxides, and automated optical/electrochemical stations can measure their PEC response in parallel. The integration of robotic synthesis with ML (the self-driving lab paradigm) could greatly accelerate photoanode development. For example, a robotic system might iterate through cycles of depositing a set of doped oxide thin films, measuring their photocurrents, and using an ML algorithm to decide the next set of dopants to try—working 24/7 without human intervention. Early demonstrations of autonomous materials labs (mostly in battery electrode or thin-film PV research) have shown significantly accelerated discovery rates. Translating this to PEC photoanodes is an engineering challenge (handling liquids, light, etc., in automation), but as those hurdles are overcome, we may see the first instances of ML not just guiding, but actively controlling, PEC experimentation.

### 7.5. Scaling and Manufacturing Considerations

Thus far, ML efforts have largely focused on maximizing performance metrics. In moving toward real applications, scalability, cost, and manufacturability will become key considerations—and ML can help here too. Future ML models might include the cost of raw materials as an input feature or even part of the objective function (e.g., maximize photocurrent and minimize cost per area). One can envision training models on manufacturing data—for instance, a model could learn from roll-to-roll produced photoanode films how uniformity and defect density (captured via inline imaging) relate to performance and yield. This could lead to ML-driven process control in factories, ensuring that scale-up does not degrade the finely tuned properties achieved in the lab. Also, ML can assist in reliability engineering by analyzing large-scale module test data to predict failures (similar to how ML monitors solar PV farm outputs). In short, as the technology matures from lab to pilot scale, ML will remain relevant at each stage: material discovery, device prototyping, and eventual manufacturing and field deployment.

### 7.6. Emerging and Future Opportunities

There are some exciting frontiers where ML could make future impacts:

- DFT + ML for reaction mechanisms: Applying ML to atomistic simulations (e.g., using neural networks to fit potential energy surfaces from DFT data) can allow simulation of complex surface reactions at lower cost. This could yield molecular-level understanding of water oxidation intermediates on photoanode surfaces. For instance, a ML-accelerated DFT study might map out how a dopant changes the binding energy of OH on a BiVO_4_ surface, explaining the catalytic effects observed.
- Inverse design via generative models: Generative adversarial networks (GANs) and other generative models can propose entirely new crystal structures or compositions optimized for target properties [65]. In the future, one might use a generative model to design a hypothetical oxide with a specific band structure and defect tolerance suitable for PEC operation—effectively inventing a new material on the computer that human experts might not conceive. Early attempts of GANs in material science have produced plausible new materials for batteries and photovoltaics; extending this to photoanodes is a matter of incorporating the right training data (perhaps from known photocatalysts).
- Knowledge incorporation: Expert knowledge and heuristics (like “d^0^ transition metal oxides tend to be good photoanodes” or “covalent oxyhydroxides have slow OER kinetics”) could be encoded as priors or constraints in ML models. This hybrid of expert systems and ML could lead to more efficient learning from smaller datasets and ensure that well-established principles guide the search, preventing the model from wasting time on chemically unreasonable candidates.
- Machine learning for co-catalyst design: We discussed catalysts in context of surfaces, but ML could also help design new water oxidation catalysts specifically tailored to photoanodes (for instance, ones that operate at lower overpotential or form ideal junctions with the semiconductor). Using datasets of electrocatalytic OER performance, ML models have started to identify descriptor-based trends. These can be applied to suggest catalysts that not only are active, but also chemically compatible with the photoanode (e.g., not leaching into it or blocking light). The ML model might learn, for example, that cobalt phosphates work well on BiVO_4_ because they passivate surface states without absorbing much light and then suggest analogous materials for other photoanodes.
- Cross-domain synergy: Combining data from photoelectrodes, photocatalytic powders, and electrocatalysts via transfer learning could create comprehensive models that understand the water splitting process in various forms. Lessons learned in one domain (e.g., stability trends in electrocatalysts) could inform predictions in another (stability of photoanodes).

In facing these challenges and opportunities, interdisciplinary collaboration will be crucial. Chemists, materials scientists, data scientists, and engineers need to work closely to ensure ML models are developed with a firm grounding in physical reality and that experimental efforts align to provide the needed data. There is also a cultural shift underway: embracing open data and sharing “failures” as well as successes, which is essential for effective machine learning. As this culture takes hold, the pace of innovation is likely to accelerate.

### 7.7. Outlook

Looking ahead 5–10 years, we anticipate a landscape where machine learning is an integral part of PEC research. A researcher designing a new photoanode might routinely use an AI assistant that suggests promising compositions or synthetic routes backed by thousands of prior experiments in its database. Computational screening for new materials will be carried out not by weeks of brute-force DFT, but by a learned model that can evaluate millions of candidates in hours [70]. In the laboratory, automated systems guided by ML will tune and re-tune photoanode samples until reaching peak performance, akin to an AI-driven optimization on the fly. And when perplexing results arise, interpretable ML will help diagnose the causes (be it a material issue or an instrument artifact).

The synergy of human intuition with machine learning’s pattern recognition and optimization prowess is especially potent in a complex, multidisciplinary problem like solar water splitting. Human researchers excel at conceptual leaps and defining the problems to solve, whereas ML excels at sifting high-dimensional data and optimizing within constraints. By leveraging both, the field stands a better chance of overcoming its long-standing hurdles, such as attaining high efficiencies without sacrificing stability or earth-abundance.

In conclusion, while challenges remain, they are surmountable through continued advancements in data generation, algorithm development, and human–machine collaboration. The rapid progress in ML algorithms and computational power, combined with an increasingly rich trove of materials data, gives confidence that many of today’s obstacles in photoanode development can be tackled in innovative ways. Machine learning is not a magic bullet, but it is a transformative tool—one that, when used judiciously, can guide us through the vast search space of materials and configurations toward practical and efficient solar fuel production. The next breakthroughs in PEC water splitting may very well emerge not from serendipity alone, but from the focused guidance of intelligent algorithms working hand-in-hand with scientists.

## 8. Conclusions

Photoelectrochemical water splitting is a grand scientific challenge at the nexus of materials science, chemistry, and renewable energy. Nanostructured metal oxide photoanodes have long been at the forefront of this research, offering a compelling combination of stability and (partially) visible-light activity, yet they have historically fallen short of the efficiency needed for practical solar fuel generation. The integration of machine learning into this field is rapidly altering the landscape. As detailed in this review, ML-driven approaches are enabling leaps in both material discovery—identifying new oxide compositions and dopants with ideal bandgaps and robust performance—and material optimization—fine-tuning known photoanodes (like TiO_2_, Fe_2_O_3_, WO_3_, BiVO_4_) to push them closer to theoretical efficiency limits. Furthermore, machine learning extends its benefits to device-level innovations, helping design multi-junction PEC systems and predict operational behavior, all while accommodating real-world considerations like stability and cost.

We highlighted how ML algorithms, from regression models and random forests to neural networks and Bayesian optimizers, have been applied to the following: (i) screen massive chemical spaces for promising photoanode materials [64,70]; (ii) unravel complex multi-parameter effects of doping, morphology, and interfaces on photoanode performance [47,67]; (iii) suggest targeted experiments that yielded record-breaking photocurrents in BiVO_4_ and other systems [49]; and (iv) aid in assembling tandem devices that achieve high solar-to-hydrogen conversion efficiencies [68]. In many cases, these ML-guided outcomes were accomplished with far fewer trials than conventional methods, underscoring the efficiency gains from data-driven research.

Crucially, we also discussed the challenges that must be addressed to fully harness ML for PEC water splitting. High-quality, standardized datasets are the fuel for the ML engine, and community efforts toward data sharing and curation will substantially enhance model capabilities. The interpretability and physical fidelity of models remain essential—progress in explainable AI and physics-informed ML is ensuring that the predictive power of algorithms comes with understanding, thus reinforcing, rather than replacing, fundamental scientific insight [65]. The eventual goal is a seamless human-AI collaboration: researchers leverage ML to explore ideas that would be prohibitively complex otherwise, and in turn, they imbue models with domain knowledge and intuition to guide the learning process.

The marriage of machine learning with experimental and theoretical PEC research is still in its formative stage, yet its impact is already evident in the accelerating pace of discovery. In a few short years, ML has helped elevate metal oxide photoanodes to new performance heights [49] and has opened avenues to explore materials (such as multinary oxides and mixed anion compounds) that were previously too complex to systematically study. The continued refinement of these approaches can be expected to yield further advances. We foresee that machine learning will become a standard component of the photoelectrode R&D toolkit—much as electrochemical workstations and solar simulators are today.

In conclusion, the integration of ML methods represents a paradigm shift in the design of nanostructured metal oxide photoanodes for water splitting. By synergistically combining materials informatics with experimental science, we are moving from a serendipity-driven mode to a predictive, “design-and-discover” mode for solar fuels materials. This shift is expediting the development of photoanodes that simultaneously meet the efficiency, stability, and cost criteria for practical hydrogen production. While significant work remains to translate lab-scale successes to scalable systems, the outlook is decidedly optimistic. If the current trajectory of ML-guided innovation continues, the coming decade could witness the realization of efficient and durable oxide-based PEC cells—devices that convert sunlight and water into clean hydrogen fuel with the aid of algorithms that have learned the secrets of materials far faster than any human. In solving the puzzle of solar water splitting, machine learning is proving to be a key that is unlocking innovation at an unprecedented rate, bringing the vision of sustainable solar fuels ever closer to reality.

## Figures and Tables

**Table 1:** Categorization of ML approaches by application stage in PEC photoanode development.

ML Approach Type	Representative Algorithms	Application Stage
Supervised Learning	Random Forests, SVM, Neural Networks	Property prediction, dopant selection, J–V modeling
Unsupervised Learning	K-means, PCA, Clustering methods	Data pattern discovery, material classification
Bayesian Optimization	Gaussian Process Regression + Acquisition Functions	Composition/morphology optimization
Physics-Informed ML	Physics-constrained Neural Networks, Hybrid DFT-ML	Predictive modeling with physical priors
Image-based Learning	CNN, Vision Transformers	Morphology-property correlation from SEM images

**Table 2:** Comparison of machine learning models in PEC photoanode applications.

ML Type	Input Features	Dataset Size	Target Property	Validation Method	Performance Metrics
CNN (image-based)	SEM images	~100 samples	Full J–V curve	Train/test split	R^2^ ≈ 0.95
ANN (structured data)	Doping level, bath pH, catalyst presence	297 samples	Photocurrent @ V_bias	K-fold CV (k = 5)	MAE ≈ 0.03 mA·cm^−2^
Random Forest	Dopant ion radius, formation energy	85 dopants	ΔPhotocurrent (Fe_2_O_3_)	Train/test split	R^2^ ≈ 0.89
Decision Tree Classifier	Bandgap, effective mass, DFT features	~1000 compounds	Activity classification	Accuracy metrics	Accuracy ≈ 81%
Random Forest	Literature-derived structure/morphology	10,000+ records	Bandgap classification	Confusion matrix	Accuracy ≈ 85%
ANN	Annealing temp, thickness, pH	~150 samples	Photocurrent profile	Bayesian optimization	Relative Error < 0.05%

## Data Availability

Data are available through request to the corresponding author.
